# Bulk-phase and interface stability strategies of manganese oxide cathodes for aqueous Zn-MnO_x_ batteries

**DOI:** 10.3389/fchem.2022.1000337

**Published:** 2022-09-23

**Authors:** Gaoqi Yang, Houzhao Wan

**Affiliations:** ^1^ Hubei Yangtze Memory Laboratories, Wuhan, China; ^2^ School of Microelectronics and Faculty of Physics and Electronics Science, Hubei University, Wuhan, China

**Keywords:** MnOx, stability, cathode, interface, zinc ion battery

## Abstract

The cyclic stability of the MnO_x_ cathodes for rechargeable zinc ion batteries have substantial obstacles due to Mn^3+^ disproportionation produces Mn^2+^ caused by Jahn Teller lattice distortion effect in the process of Zn^2+^ inter/deintercalation. This mini review summarized bulk-phase and interface stability strategies of manganese oxide cathodes for aqueous Zn-MnO_x_ batteries from the regulation of bulk electronic state of manganese oxide improves its structural stability and the formation of beneficial SEI layer at the interface of electrolyte. It provides theoretical support for the design of manganese oxide cathode materials for aqueous zinc ion batteries with high stability.

## Introduction

Human society has been seeking new energy storage equipment with higher performance (such as higher energy density, power density and long life), low cost and high security to alleviate outstanding environmental problems and severe energy situation (Pomerantseva et al., 2019; Chen et al., 2021; Zheng et al., 2021). At present, lithium-ion batteries are widely used in mobile devices because of their high energy/power density and long life (Manthiram, 2020; Chen et al., 2022). However, with the increasing demand for large-scale energy storage such as electric vehicles and smart grid energy storage systems, as well as the high price of lithium resources and security, other high-performance and low-cost energy storage devices are needed to supplement (Jia et al., 2020; Zhong et al., 2020; Zhou et al., 2020). In recent years, batteries based on other monovalent metal ions (Na^+^, K^+^) and multivalent metal ions (Ca^2+^, Mg^2+^, Zn^2+^, Al^3+^, etc.) have attracted extensive attention due to their rich content and low cost (Li et al., 2020; Pan et al., 2021; Zhou and Guo, 2021). The aqueous zinc ion batteries used zinc metal as the anode material, which has many advantages, such as high theoretical specific capacity (820 mAh g^−1^), low oxidation-reduction potential of Zn^2+^/Zn(−0.76 V relative to SHE), which endows the aqueous zinc ion battery with the intrinsic characteristics of environmental friendliness, low cost and high safety (Cui et al., 2022). Especially, zinc ion battery as a kind of multivalent ion batteries, its energy density tends to increase due to the presence of high-capacity multivalent metals as the anode (Selvakumaran et al., 2019). The volumetric energy density of zinc ion battery is as high as 5851 mAh cm^−3^ higher than Li^+^ (2061 mAh cm^−3^), Mg^2+^ (3834 mAh cm^−3^), Ca^2+^ (2072 mAh cm^−3^) (Chen et al., 2019). This makes the aqueous rechargeable zinc ion battery one of the most promising electrochemical energy storage devices.

Because zinc metal anode has many advantages, the development of aqueous rechargeable zinc ion battery is largely limited by the cathode materials (Xia et al., 2018; Liu et al., 2019). At present, manganese based materials (MnO_2_, Mn_3_O_4_, MnO, etc.) (Tang et al., 2019; Zhu et al., 2020), vanadium based materials (V_2_O_5_, VO_2_, Zn_0.25_V_2_O_5_, etc.) (Wan and Niu, 2019; Boruah et al., 2021; Wang et al., 2022) and Prussian blue (Ming et al., 2019) have been studied as cathode materials for aqueous Zn ion batteries. Among them, manganese oxides (MnO_x_) have many advantages, such as high operating voltage (∼1.35 V), high theoretical specific capacity (based on single electron reaction ∼308 mAh g^−1^), multiple valence states of manganese, low cost, etc., making MnO_x_ considered as one of the most potential cathode materials for aqueous rechargeable zinc ion batteries (Wang et al., 2020; Li et al., 2022; Zhang et al., 2022). These outstanding advantages make MnO_x_, including α-, β-,γ-, λ-, δ- MnO_2_, MnO, Mn_3_O_4_ and other structures are sought after. In 2015, S. H. Oh reported that α-MnO_2_ (2 × 2 channels) showed a specific capacity of 195 mAh g^−1^ at 10 mA g^−1^ and maintained 70% after 30 cycles (Lee et al., 2015). Although MnO_x_ cathode materials show considerable specific capacity, they face the problem of rapid degradation of cycle stability. In order to solve this problem, Y.Y. Xia and Y.G. Wang inserted polyaniline into layered MnO_2_, which improved the stability of the structure and improved the cycle stability (Huang et al., 2018). J. Zhou and S.Q. Liang grew Mn_3_O_4_ *in situ* on stainless steel mesh, and the structure maintained 500 cycles at 500 mA g^−1^ (Zhu et al., 2018). These measures can help to improve the battery stability, but there are still substantial obstacles to the application of MnO_x_ based rechargeable zinc ion battery cathode materials (Ming et al., 2019): 1) Mn^3+^ disproportionation produces Mn^2+^ caused by Jahn Teller (J-T) lattice distortion effect in the process of Zn^2+^ inter/deintercalation, which will directly lead to the decline of MnO_x_ cycle stability. 2) The wide band gap of MnO_x_ leads to low conductivity, which results in its unsatisfactory rate capability.

In this mini review, the stability and rate capability improvement strategies of manganese oxide cathode materials are proposed from two perspectives: 1) The electronic state structure of MnO_x_ is regulated through defect engineering (O/Mn vacancy and transition metal doping), which can improve its structural stability, band gap and electrochemical activity, to inhibit Mn dissolution caused by J-T effect, accelerate ion/electron conduction, and improve cycle stability and rate capability. 2) The interfacial reaction kinetics of MnO_x_ cathode and electrolyte are optimized enriching with electrolyte additives, which can be inhibit the dissolution of Mn in the process of MnO_x_ zinc storage and accelerate the diffusion kinetics of ion interface, so as to effectively improve the cycle stability and specific capacity. This review provides theoretical support for the design of manganese oxide cathode materials for aqueous zinc ion batteries with high stability.

## Bulk-phase regulation

**FIGURE 1:**
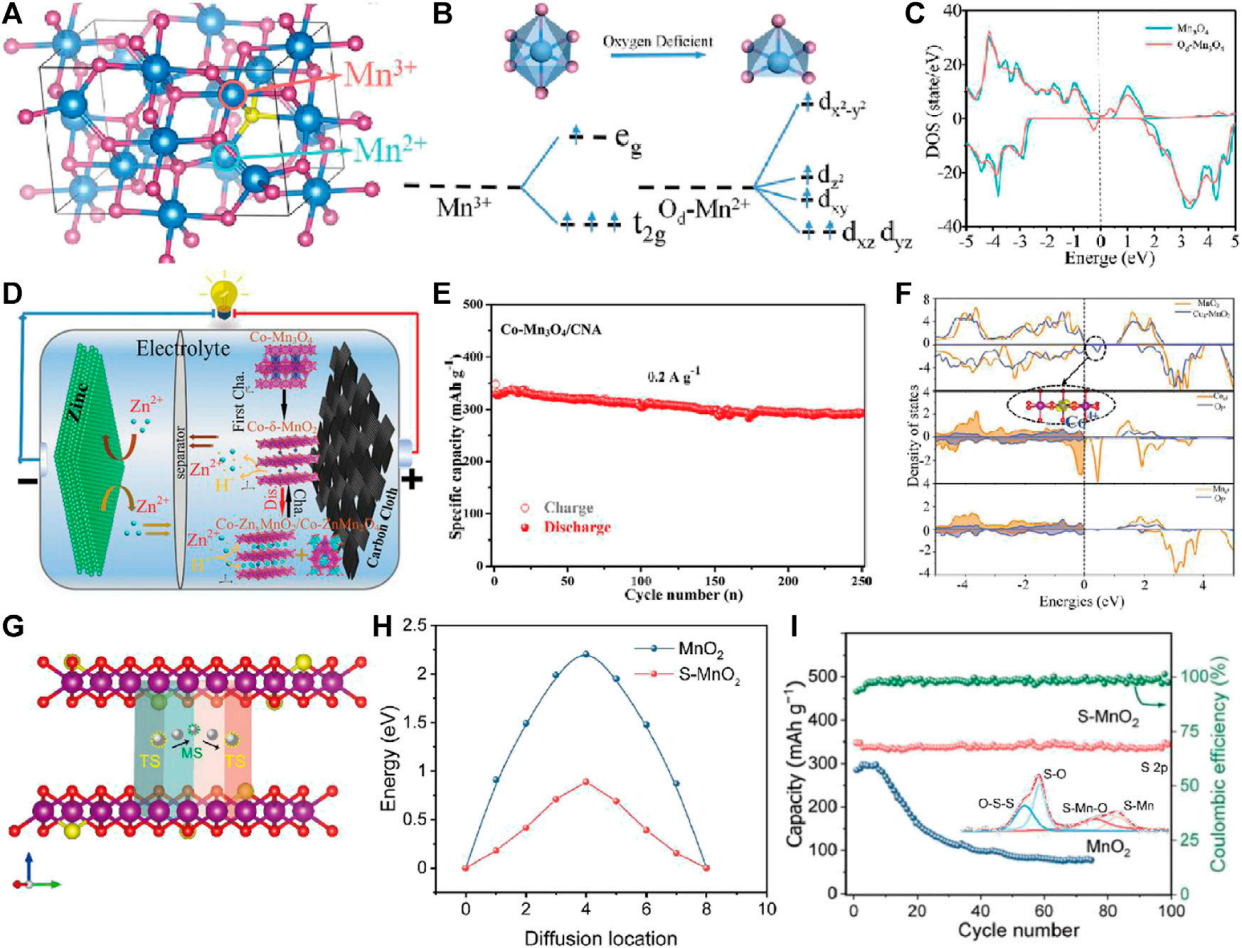
**(A)** Supercell model of Mn_3_O_4_. **(B)** The Mn-O octahedral and pyramidal crystal fields and the d-orbital splitting configurations. **(C)** The TDOS of the Mn_3_O_4_ and Od-Mn_3_O_4_ bulk phase. **(D)** Schematic illustration of Zn//Co-Mn_3_O_4_/CNA battery. **(E)** Cycling performance of the Co-Mn_3_O_4_/CNA at 0.2 A g^−1^. **(F)** DOS of MnO_2_ and Co^4+^-δ-MnO_2_. **(G)** The side view of schematic illustration of Zn migration in S-MnO_2_. **(H)** The Zn ion diffusion barrier profiles for MnO_2_ and S-MnO_2_. **(I)** Cycling performance of MnO_2_ and S-MnO_2_ electrodes at 0.2 A g^−1^.

By changing the electronic state structure of t_2_g^3^egfn1 with high spin of Mn^3+^ and substituting transition metal elements for Mn^3+^, the structural stability of MnO_x_ cathode material is improved to suppress the J-T effect, so as to improve its cycle stability, which has been deeply studied in the application of lithium/sodium ion batteries (Li et al., 2009; Xiao et al., 2018). Defect engineering (such as vacancy and doping) can improve the structural stability of MnO_x_, inhibit Mn^3+^ disproportionation caused by J-T effect, and improve the cycle stability of MnO_x_ zinc storage process (H Zheng et al., 2022; Ji et al., 2022). At present, part of the work has carried out defect engineering research on manganese-based cathode materials for aqueous zinc ion batteries. In 2019, J. M. Xue pointed out that the Gibbs free energy of Zn^2+^ adsorption in the vicinity of O vacancy can be reduced to thermoneutral value (≈0.05 eV) by introduction of O vacancy in MnO_2_ lattice, which suggested that Zn^2+^ adsorption/desorption process on oxygen-deficient MnO_2_ is more reversible (Xiong et al., 2019). L. Q. Mai Doped Ti by surface gradient α-MnO_2_ produces electron compensated O vacancy, which accelerates the migration rate of ions/electrons, thus improving the diffusion coefficient of Zn^2+^/H^+^ in Ti-MnO_2_ (Lian et al., 2019). However, it mainly focuses on improving the electron/ion transfer rate to improve its performance, and less attention is paid to the influence of defect states on the electronic structure of Mn^3+^ and the improvement of the structural stability of MnO_x_ to inhibit J-T from improving its zinc storage cycle stability. H. Wang and H. Z. Wan introduced O vacancy into Mn-O octahedron of Mn_3_O_4_ to improve the electronic structure of Mn_3_O_4_, thus improving the cycle stability (Figures 1A–C) (Tan et al., 2020). At the same time, they have effectively improved the structural stability of Mn_3_O_4_ and realized high cycle stability by replacing Mn with Co^2+^ (Figures 1D–F) (Ji et al., 2021). The sulfur doped MnO_2_ (S-MnO_2_) nanosheets have been found to be high-performance cathodes for zinc ion batteries (Figures 1G–I). The doped S atoms in O sites with lower electronegativity can improve its bulk conductivity, reduce the electrostatic interaction with Zn^2+^, and accelerate the reaction kinetics to improve cycle stability (Zhao et al., 2022). The MnO_x_ cathode can be modified by defect engineering (O/Mn vacancy or transition metal element doping, etc.) to improve structural stability, inhibit Mn dissolution, and effectively improve cycle stability. This has important theoretical significance for the commercialization of manganese based zinc ion batteries.

## Electrode/electrolyte interface optimization

**FIGURE 2:**
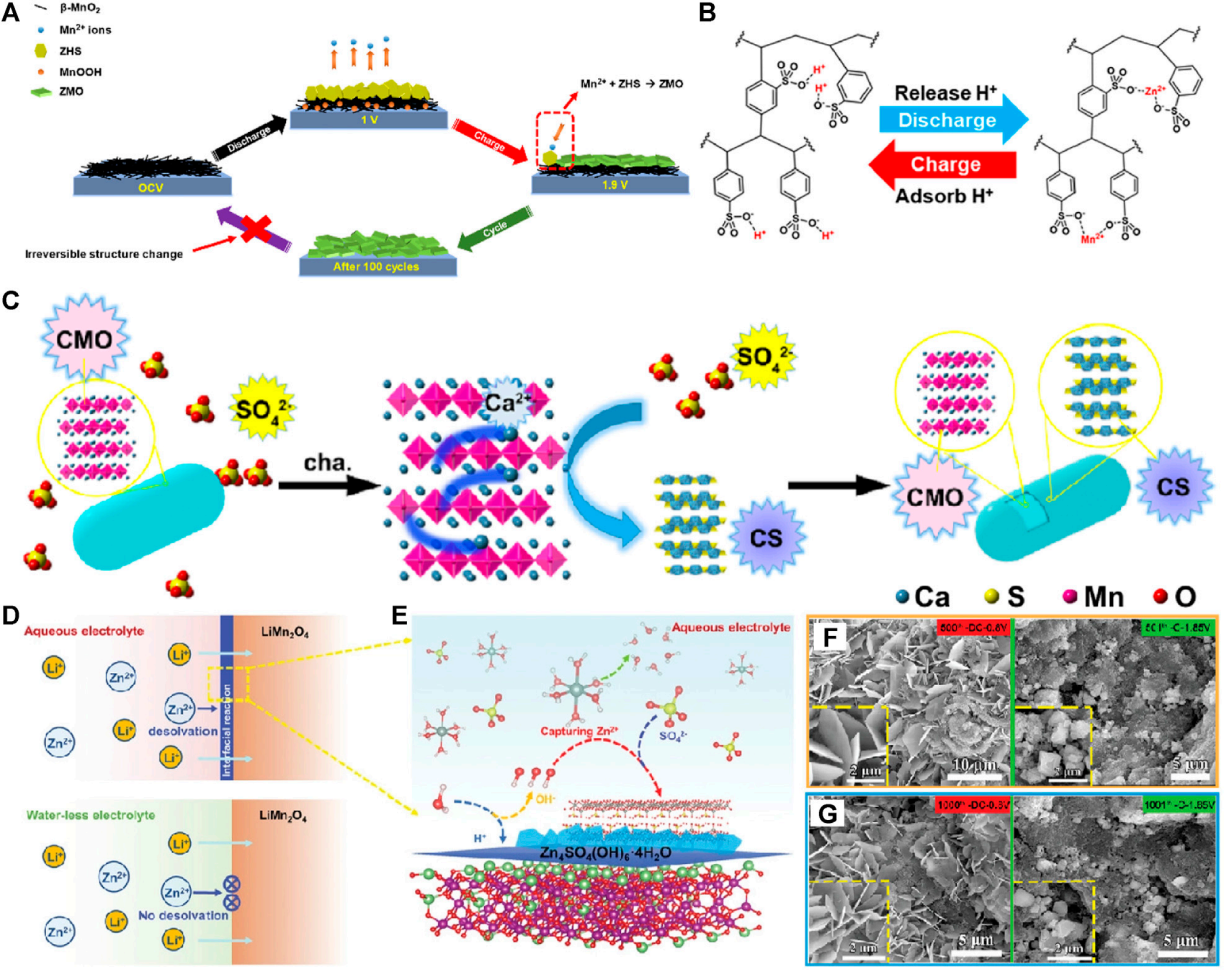
**(A)** A schematic illustration of the irreversible structure change on the β-MnO_2_ cathode. **(B)** Schematic illustration of IER releasing and adsorbing protons. **(C)** The formation mechanism of the unique structure of CaSO_4_·2H_2_O (CS) SEI layer-coated Ca_2_MnO_4_ (CMO). **(D)** Schematic illustration of cathode/electrolyte interface in aqueous and waterless electrolyte. **(E)** The working mechanism of the interfacial reaction of the LiMn_2_O-cathode in ZnSO_4_-based aqueous electrolyte. **(F)** The *ex-situ* SEM images at fully discharged/charged state after **(G)** the 500th cycle and **(E)** the 1000th cycle.

The electrode/electrolyte interface reaction process plays a key role in the performance of electrochemical energy storage devices. The different types of electrodes to optimize the electrode/electrolyte interface reaction dynamics using advanced electrolyte and new electrolyte additives can maximize the performance of aqueous zinc ion batteries (Wang et al., 2018; Xu et al., 2019). In 2016, J. Liu added Mn^2+^ into 2 M ZnSO_4_ electrolyte to improve the Mn^2+^ dissolution chemical potential of α-MnO_2_ electrode material in the process of zinc storage, which can effectively inhibit the J-T effect and improves cycle stability of α-MnO_2_/Zn battery (Pan et al., 2016). P. Chen etc (Wu et al., 2022) reported that the by-product of Zn4(OH)6SO4∙xH2O (ZHS) from pH fluctuation can react with Mn^2+^ during charge process at over 1.55 V to form a lowly crystallized (Zn,Mn)2Mn5O12∙4H2O (ZMO), which severely hinders the capacity of MnO_2_ cathode rapid fading (Figures 2A,B). The introduction of ion exchange resin (IER) can adjust the proton distribution in the electrolyte and eliminate the above adverse ZHS, so as to effectively inhibit the formation of ZMO, to improve cycle stability of MnO_2_ as cathode of Zn/MnO_2_ battery. Although some studies have shown that electrolyte optimization can improve the cycle stability of MnO_x_ cathode materials, it is difficult to ensure that the cycle stability is improved without loss of specific capacity and rate performance.

In addition, the adverse effects of zinc hydroxysulfate by-products produced at the electrode/electrolyte interface of Mn based cathode materials in the process of Zn^2+^/H^+^ co-insertion have not attracted much attention. The interface of the MnOx electrode/electrolyte form a layer of zinc hydroxysulfate favorable SEI film from adverse factors by adjusting electrolyte environment, which can inhibit the dissolution of Mn^2+^, and effectively improve the cycle stability and the specific capacity and rate performance at the same time. J. Zhou found in the research of Ca_2_MnO_4_/Zn battery containing (Zn^2+^+Mn^2+^)SO_4_^2-^ electrolyte that CaSO_4_·2H_2_O-SEI film formed on the surface of Ca_2_MnO_4_ cathode material, which effectively inhibited the dissolution of Mn during charge and discharge, and significantly improved the stability of the battery compared with the battery containing only CH_3_COO electrolyte (Figure 2C) (Shan et al., 2019). G.Z. Fang and S.Q. Liang put forward the relationship between the desolvation of Zn^2+^ from [Zn(OH_2_)_6_]^2+^-solvation shell and the electrolyte/electrode interfacial reaction to form Zn_4_SO_4_(OH)_6_·4H_2_O phase (Figures 2D–G). The Zn//MnO_2_ battery based on electrolyte optimization displayed the cycling stability of over 2000 cycles (Zhang et al., 2020). Thus, the process dynamics of the interface between cathode and electrolyte can be optimized by adjusting the electrolyte additives to match the manganese removal cathode materials, and the cycle stability can be effectively improved.

## Conclusion and perspectives

This work summarizes the bulk-phase and interface stability strategies of manganese oxide cathodes for aqueous Zn-MnO_2_ batteries. On the one hand, defect engineering regulated MnO_x_ cathode can improve its structural stability and conductivity to improve cycle stability and rate capability. On the other hand, the interfacial reaction kinetics of MnO_x_ cathode and electrolyte are optimized enriching with electrolyte additives can inhibit the dissolution of Mn and accelerate the diffusion kinetics of ion interface to effectively improve the cycle stability and specific capacity.

Although a lot of research work has been done on the optimization and modification of the cycle stability of MnO_x_ cathode materials, the research on the regulation of bulk electronic states and the mechanism of interface adaptation is not deep and systematic, and the cycle stability that meets the commercial application has not been completely solved. To further improve the overall performance of MnO_x_ cathode in aqueous solution system, we put forward the following anticipations: 1) To explore the best synthetic means and defect types of bulk electronic state regulation of manganese oxide, and systematically study the internal mechanism of bulk electronic state regulation; 2) The Zn^2+^ solvation structure was optimized by adding polar molecules or other additives into the electrolyte to improve the interface structure of manganese oxide and electrolyte. 3) Seeking an electrolyte adaptation system for *in-situ* construction of favorable SEI film on the surface of MnO_x_ cathode, and establish a theoretical system of interface optimization; 4) Study the influence of parameters such as cathode and anode matching, electrolyte proportion and adaptation on device performance, and carry out a comprehensive evaluation of key performance such as energy density, power density and cycle stability of aqueous MnO_x_/Zn battery.
